# Nucleotide Pyrophosphatase/Phosphodiesterase 1 Exerts a Negative Effect on Starch Accumulation and Growth in Rice Seedlings under High Temperature and CO_2_ Concentration Conditions

**DOI:** 10.1093/pcp/pct139

**Published:** 2013-10-21

**Authors:** Kentaro Kaneko, Takuya Inomata, Takahiro Masui, Tsutomu Koshu, Yukiho Umezawa, Kimiko Itoh, Javier Pozueta-Romero, Toshiaki Mitsui

**Affiliations:** ^1^Department of Applied Biological Chemistry, Niigata University, 2-8050 Ikarashi, Niigata, 950-2181 Japan; ^2^Graduate School of Science and Technology, Niigata University, 2-8050 Ikarashi, Niigata, 950-2181 Japan; ^3^Instituto de Agrobiotecnología (CSIC, UPNA, Gobierno de Navarra). Mutiloako etorbidea zenbaki gabe, 31192 Mutiloabeti, Nafarroa, Spain; ^4^These authors contributed equally to this work.

**Keywords:** ADP-glucose, CO_2_, NPP, *Oryza sativa*, Plastid, Starch

## Abstract

Nucleotide pyrophosphatase/phosphodiesterase (NPP) is a widely distributed enzymatic activity occurring in both plants and mammals that catalyzes the hydrolytic breakdown of the pyrophosphate and phosphodiester bonds of a number of nucleotides. Unlike mammalian NPPs, the physiological function of plant NPPs remains largely unknown. Using a complete rice NPP1-encoding cDNA as a probe, in this work we have screened a rice shoot cDNA library and obtained complete cDNAs corresponding to six NPP genes (*NPP1–NPP6*). As a first step to clarify the role of NPPs, recombinant NPP1, NPP2 and NPP6 were purified from transgenic rice cells constitutively expressing *NPP1*, *NPP2* and *NPP6*, respectively, and their enzymatic properties were characterized. NPP1 and NPP6 exhibited hydrolytic activities toward ATP, UDP-glucose and the starch precursor molecule, ADP-glucose, whereas NPP2 did not recognize nucleotide sugars as substrates, but hydrolyzed UDP, ADP and adenosine 5′-phosphosulfate. To gain insight into the physiological function of rice NPP1, an *npp1* knockout mutant was characterized. The ADP-glucose hydrolytic activities in shoots of *npp1* rice seedlings were 8% of those of the wild type (WT), thus indicating that NPP1 is a major determinant of ADP-glucose hydrolytic activity in rice shoots. Importantly, when seedlings were cultured at 160 Pa CO_2_ under a 28°C/23°C (12 h light/12 h dark) regime, *npp1* shoots and roots were larger than those of wild-type (WT) seedlings. Furthermore, the starch content in the *npp1* shoots was higher than that of WT shoots. Growth and starch accumulation were also enhanced under an atmospheric CO_2_ concentration (40 Pa) when plants were cultured under a 33°C/28°C regime. The overall data strongly indicate that NPP1 exerts a negative effect on plant growth and starch accumulation in shoots, especially under high CO_2_ concentration and high temperature conditions.

The nucleotide sequence reported in this paper has been submitted to the DDJB with accession number AB196673.

## Introduction

Nucleotide pyrophosphatase/phosphodiesterases (NPPs) are widely distributed *N*-glycosylated enzymes that catalyze the hydrolytic breakdown of the pyrophosphate and phosphodiester bonds of numerous nucleotides and nucleotide sugars (Rodríguez-López et al. 2000, Nanjo et al. 2006, Kaneko et al. 2011). Unlike nucleotide hydrolases of the Nudix family (McLennan 2006) and diphosphonucleotide phosphatase/phosphodiesterases (Olczak et al. 2009), plant NPPs do not require metal ions to be fully active (Rodríguez-López et al. 2000, Nanjo et al. 2006). NPPs are predicted to have endomembrane system localization, as confirmed by confocal microscopy analyses of NPP3 fused with green fluorescent protein (GFP) (Kaneko et al. 2011). However, immunocytochemical studies of rice cells using polyclonal anti-NPP1 antibodies as well as confocal laser scanning microscopic analyses of rice cells expressing NPP1 fused with GFP provided strong evidence that NPP1 is localized in the plastidic compartment (Nanjo et al. 2006). Moreover, treatment with brefeldin A, a potent inhibitor of Golgi–endoplasmic reticulum (ER) vesicle trafficking, prevents NPP1–GFP targeting to chloroplasts of rice cells (Nanjo et al. 2006). The overall data thus strongly indicated that, similarly to other proteins that are predicted to reside in the endomembrane system (Chen et al. 1994, Chen et al. 2004, Asatsuma et al. 2005, Villarejo et al. 2005, Kitajima et al. 2009, Burén et al. 2011), NPP1 is transported from the ER–Golgi system to the chloroplast compartment through the secretory pathway in rice cells (Nanjo et al. 2006).

Mammalian NPPs have been shown to be involved in a variety of cellular processes such as nucleotide signaling, cell differentiation, nucleotide recycling and control of the levels of nucleotides linked to glycosylation and sulfation reactions (Hickman et al. 1985, Goding et al. 1998, Frittitta et al. 1999, Bollen et al. 2000, Lazarowski et al. 2000, Gisjsbers et al. 2001). In clear contrast, practically nothing is known about the role of NPPs in plants, although previous studies have suggested that NPPs could be involved in the fine regulation of metabolic flux towards starch biosynthesis by controlling the intracellular concentrations of the starch precursor molecule, ADP-glucose (Rodríguez-López et al. 2000). As a first step to investigate the role of NPPs, in this work we have characterized the enzymatic properties of recombinantly produced NPP1, NPP2 and NPP6. These studies revealed that NPP1 exhibits the highest hydrolyzing activity toward ADP-glucose, ADP-ribose, ATP and UDP-glucose. Subsequent studies using *npp1* null mutants cultured under different temperature and CO_2_ concentration conditions strongly indicated that NPP1 exerts a negative effect on plant growth and starch accumulation, especially under high CO_2_ concentration conditions, which provides the first in vivo evidence about the possible involvement of NPP1 in the control of plant growth and reserve carbohydrate accumulation in rice under high CO_2_ concentration conditions.

## Results and Discussion

### Identification of the NPP gene family members

**Fig. 1:**
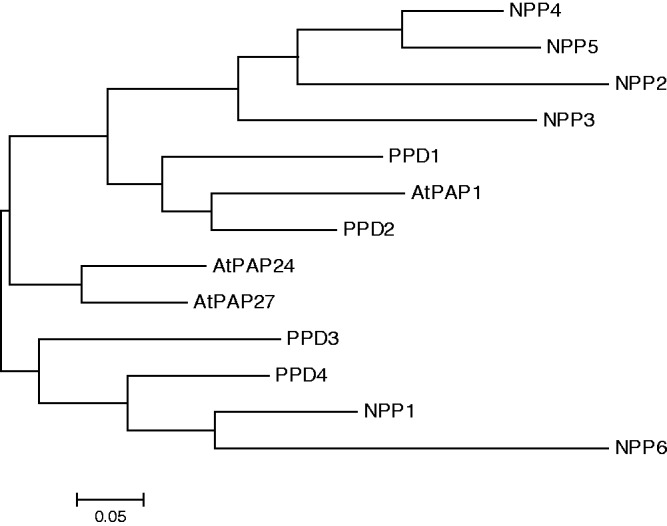
NPP family. The multiple sequence alignment was made using the ClustalW program with MEGA5. Sequences used were those deduced from rice *NPP1–NPP6*, yellow lupin *PPD1–PPD4* and Arabidopsis *PAP1*, *PAP24* and *PAP27*. The scale bar shows the number of substitutions per site.

cDNAs corresponding to six different NPP-encoding genes (*NPP1–NPP6*) were obtained from rice shoot cDNA libraries using an NPP1-encoding cDNA (Nanjo et al., 2006) as a probe. The six genes are registered in the Oryzabase (http://www.shigen.nig.ac.jp/rice/oryzabase_submission/gene_nomenclature/; McCouch, 2008), *NPP2* being a gene whose sequence had not previously been deposited in public databases. *NPP2* cDNA is 2,347 bp in length and includes a single open reading frame of 1,854 bp that encodes 617 amino acid residues, with a predicted molecular mass of 69.6 kDa and a pI of 6.08. The open reading frames of *NPP1–NPP6* cDNA encode proteins with molecular masses of 68.1–69.9 kDa and a pI of 5.85–6.20. *NPP* genes were located on chromosomes 3 (*NPP3*), 8 (*NPP1*), 9 (*NPP6*) and 12 (*NPP2*, *4* and *5*) in rice (see Supplementary Table S1). A comparison of the deduced amino acid sequences of proteins using ClustalW (http://align.genome.jp/) showed that (i) the degrees of similarity between NPP1 and other NPPs ranged between 61.1 and 71.6%, and (ii) 53.7% of the amino acids of NPP1, 2 and 6 are identical. NPP amino acid sequences are highly similar to those of metallophosphatases from yellow lupin (Olczak and Olczak 2002) and purple acid phosphatases 1, 24 and 27 from Arabidopsis (Zhu et al. 2005) (Fig. 1). The overall data thus indicate that NPPs belong to a large family of structurally related nucleotide hydrolases.

### Enzymatic characterization of NPP1, NPP2 and NPP6

Previous studies using recombinantly produced NPP3 revealed that this enzyme hydrolyzes several nucleoside diphosphates, but not nucleotide-sugars such as ADP-ribose, ADP-glucose or UDP-glucose (Kaneko et al. 2011). To carry out substrate specificity and kinetic studies of NPP1, NPP2 and NPP6, in this work we produced transgenic rice cells ectopically expressing *NPP1*, *NPP2* and *NPP6* and purified the corresponding recombinant proteins as described in the Materials and Methods. Details of the processes for purification of recombinant proteins and quality of the final preparations are shown in Supplementary Table S2 and Supplementary Fig. S1A.

Gel filtration analyses of the purified recombinant NPP1, NPP2 and NPP6 revealed that, in the native stage, they elute as 251, 374 and 224 kDa proteins, respectively (Table 1, Supplementary Fig. S2A). SDS–PAGE and subsequent staining of the purified NPPs revealed a single polypeptide band in each preparation, strongly indicating that NPP1, NPP2 and NPP6 form homo-oligomeric structures composed of 70, 71 or 74 kDa polypeptides, respectively (Supplementary Fig. S1B). This, and the fact that rice NPPs exhibit no requirement for a metal ion (Nanjo et al. 2006), indicates that NPPs have structural characteristics that are different from those of plant purple acid phosphatases (Beck et al. 1986, Durmus et al. 1999, Schenk et al. 2001, Waratrujiwong et al. 2006, Kaida et al. 2008, Olczak et al. 2009).

**Table 1:** Enzymatic properties of NPP1, NPP2 and NPP6

Characteristics	NPP1	NPP2	NPP6
Molecular sizes			
SDS–PAGE (gel filtration)	70 kDa (251 kDa)	71 kDa (374 kDa)	74 kDa (224 kDa)
Substrate specificity relative value, %)	ADP-glucose (100)	UDP (100)	ADP (100)
	ADP-ribose (99)	ADP (87)	UDP (68)
	TDP-glucose (82)	APS (81)	ADP-glucose (65)
	ATP (81)	CDP (56)	PPi (59)
	UDP-glucose (77)	IDP (48)	Bis-*p*-NPP (35)
	CDP-glucose (77)	TDP (40)	APS (31)
	GDP-mannose (76)	GDP (38)	TDP (27)
	APS (44)	Bis-*p*-NPP (32)	UDP-glucose (25)
	ADP (40)	ADP-glucose (NQ*^a^*)	TDP-glucose (15)
Optimum pH	pH 6.0 (ADP-glucose)	pH 4.5 (ADP)	pH 4.0 (ADP)
			pH 5.0 (ADP-glucose)
Optimum temperature	60°C	60°C	55°C
Metal ion requirement	None	None	None

The molecular sizes of purified NPP1, NPP2 and NPP6 proteins were estimated by SDS–PAGE and gel filtration chromatography with molecular weight standards as described in the Materials and Methods. The data presented in Table 2 are used to summarize substrate specificity. The maximal value of *k*_cat_*/K*_m_ for each NPP enzyme was normalized at 100%. The temperature and pH dependency of the enzyme reaction were determined using ADP-glucose and ADP as substrates. Details of temperature and pH dependency are presented in Supplementary Fig. S2.

APS, adenosine 5′-phosphosulfate; Bis-*p*-NPP, bis(*p*-nitrophenyl) phosphate; NQ, not quantifiable; PPi, pyrophosphate.

Substrate specificity and kinetic analyses revealed clearly distinguishable characteristics between NPP1, NPP2 and NPP6 (Tables 1, 2). Thus, whereas NPP1 and NPP6 catalyze the hydrolytic breakdown of nucleotide sugars such as ADP-glucose, UDP-glucose and ADP-ribose, NPP2 did not recognize these compounds as substrates, but favored the hydrolysis of UDP (*k*_cat_/*K*_m_ of 0.84 × 10^5^ s^−1^ M^−1^), ADP (*k*_cat_/*K*_m_ of 0.73 × 10^5^ s^−1^ M^−1^) and adenosine 5′-phosphosulfate (APS; *k*_cat_/*K*_m_ of 0.68 × 10^5^ s^−1^ M^−1^), a characteristic similar to that of NPP3 (Kaneko et al. 2011). NPP1 hydrolyzed ADP-glucose (*k*_cat_/*K*_m_ of 2.50 × 10^5^ s^−1^ M^−1^), ADP-ribose (*k*_cat_/*K*_m_ of 2.48 × 10^5^ s^−1^ M^−1^), TDP-glucose (*k*_cat_/*K*_m_ of 2.06 × 10^5^ s^−1^ M^−1^) and UDP-glucose (*k*_cat_/*K*_m_ of 1.93 × 10^5^ s^−1^ M^−1^) the best, while ADP and ADP-glucose were the best substrates for NPP6 (*k*_cat_/*K*_m_ of 2.97 × 10^5^ s^−1^ M^−1^ and 1.93 × 10^5^ s^−1^ M^−1^, respectively). NPP1, NPP2 and NPP6 did not recognize phosphomonoester bond-containing compounds such as sugar phosphates or nucleoside monophosphates (Table 2), and displayed a broad optimal pH range and relatively high optimal temperature for enzymatic activity (Table 1, Supplementary Fig. S2B, C).

**Table 2:** Kinetic parameters of NPP1, NPP2 and NPP6

Substrates	NPP1				NPP2				NPP6			
	*K*_m_ (mM)	*V*_max_ (U mg^−1^)*^a^*	*k*_cat_ (s^−1^)	*k*_cat_/*K*_m_ (s^−1^ M^−1^)	*K*_m_ (mM)	*V*_max_ (U mg^−1^)	*k*_cat_ (s^−1^)	*k*_cat_/*K*_m_ (s^−1^ M^−1^)	*K*_m_ (mM)	*V*_max_ (U mg^−1^)	*k*_cat_ (s^−1^)	*k*_cat_*/K*_m_ (s^−1^ M^−1^)
ADP-glucose	0.8	183	214	2.50 × 10^5^	NQ	NQ	NQ	NQ	0.7	105	130	1.93 × 10^5^
ADP-ribose	1.4	317	370	2.48 × 10^5^	NQ	NQ	NQ	NQ	NT	NT	NT	NT
GDP-mannose	0.8	129	151	1.90 × 10^5^	NQ	NQ	NQ	NQ	NT	NT	NT	NT
CDP-glucose	0.9	107	125	1.93 × 10^5^	NQ	NQ	NQ	NQ	NT	NT	NT	NT
UDP-glucose	1.5	242	282	1.93 × 10^5^	NQ	NQ	NQ	NQ	1.6	93	115	0.73 × 10^5^
TDP-glucose	NT*^b^*	NT	124	2.06 × 10^5^	NT	NT	NT	NT	1.8	65	81	0.45 × 10^5^
ATP	2.1	356	415	2.03 × 10^5^	NT	NT	NT	NT	NT	NT	NT	NT
ADP	4.3	385	449	1.00 × 10^5^	1.1	68	80	0.73 × 10^5^	0.3	68	83	2.97 × 10^5^
APS	3	282	329	1.09 × 10^5^	0.5	29	34	0.68 × 10^5^	0.8	6	76	0.93 × 10^5^
UDP	NT	NT	NT	NT	1.2	85	101	0.84 × 10^5^	0.5	84	104	2.03 × 10^5^
GDP	NT	NT	NT	NT	0.9	24	28	0.32 × 10^5^	NT	NT	NT	NT
IDP	NT	NT	NT	NT	2.2	75	88	0.40 × 10^5^	NT	NT	NT	NT
CDP	NT	NT	NT	NT	1.9	76	89	0.47 × 10^5^	NT	NT	NT	NT
TDP	NT	NT	NT	NT	2.6	74	87	0.34 × 10^5^	1.1	71	87	0.81 × 10^5^
NAD^+^	0.8	93	109	1.33 × 10^5^	NT	NT	NT	NT	NT	NT	NT	NT
NADP^+^	1.6	121	141	0.85 × 10^5^	NT	NT	NT	NT	NT	NT	NT	NT
PPi	NT	107	125	NT	2.2	34	40	0.18 × 10^5^	0.5	64	79	1.75 × 10^5^
Bis-*p*-NPP	1.3	116	223	1.71 × 10^5^	2.6	60	71	0.27 × 10^5^	3.0	250	308	1.03 × 10^5^
Hexose-P	NQ*^c^*	NQ	NQ	NQ	NQ	NQ	NQ	NQ	NQ	NQ	NQ	NQ
Mononucleotide	NQ	NQ	NQ	NQ	NQ	NQ	NQ	NQ	NQ	NQ	NQ	NQ

The enzyme assay was carried out at a substrate concentration of 0.1–10 mM, at 37°C, and at optimal pH. The data give the averages of duplicate experiments.

*^a^* U mg^−1^, µmol min^−1^ (mg of protein)^−1^.

*^b^* NT, not tested.

*^c^* NQ, not quantifiable.

### Expression profiles of *NPP1*, *NPP2* and *NPP6*

**Fig. 2:**
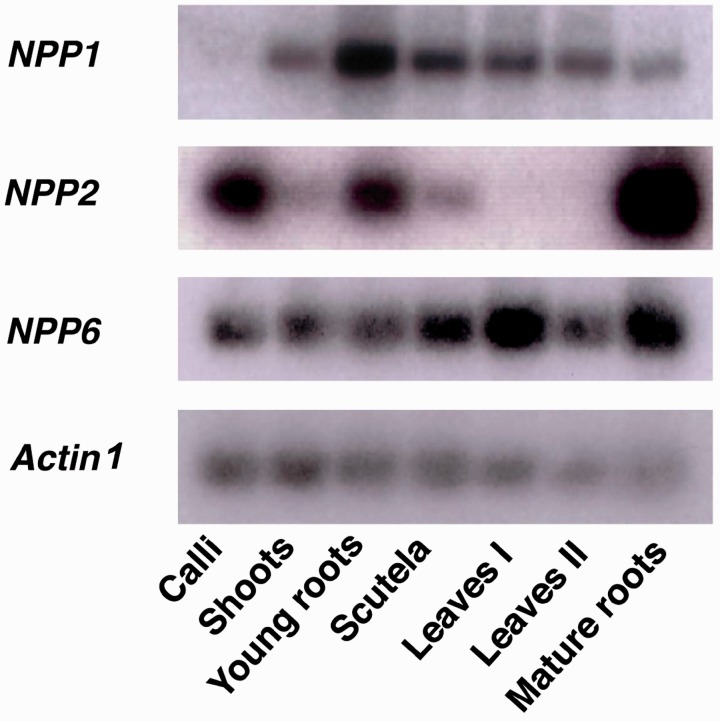
Expression profile of *NPP1*, *NPP2* and *NPP6* in rice plant. Tissue-specific distribution of *NPP1*, *NPP2* and *NPP6* transcripts. Shoots, young roots and scutella were harvested from seedlings at 7 d after imbibition (DAI). Leaves I and mature roots were harvested from plants at 1 week before heading. Leaves II were harvested from plants at 1 week after heading. Actin 1 was used as an internal control.

Tissue- and stage-specific expression of *NPP1*, *NPP2* and *NPP6* in rice plant was examined by Northern blot analyses. As shown in Fig. 2, *NPP1* was widely expressed in shoots, young roots, scutella, mature leaves and roots, but not in calli. *NPP6* was expressed in the whole plant and also in calli. In clear contrast, *NPP2* was predominantly expressed in roots (Fig. 2).

### Construction of an *npp1* null mutant

**Fig. 3:**
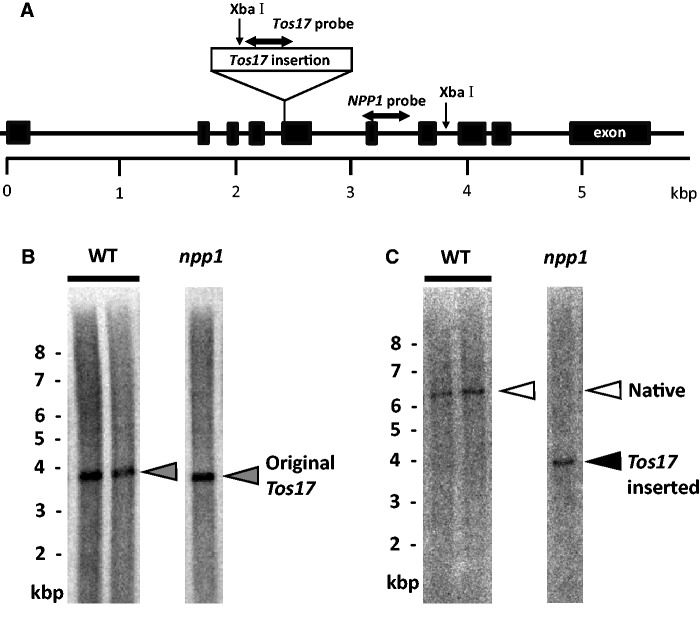
Southern blot analysis of a homozygous *Tos17*-inserted *NPP1* mutant and WT plants. (A) Diagram of the *Tos17* insertion mutation of the *NPP1* gene located on chromosome 8. The diagram illustrates the insertion site of a retrotransposon *Tos17* into exon 5 of *NPP1* of the *npp1* mutant. The exons of the *NPP1* gene are shown as black boxes. Single-headed arrows indicate the positions of the restriction sites of *Xba*I. Sequence regions indicated by horizontal double-headed arrows were used as *Tos17* and *NPP1* detection probes in Southern blot analysis. (B, C) Genomic DNA prepared from the WT and *npp1* mutant was digested with *Xba*I, followed by Southern blot analysis with the *Tos17* (B) or *NPP1* (C) probe. A gray arrowhead shows the original position of the *Tos17* gene occurring in the WT plant. White and black arrowheads indicate the presence of native and *Tos17*-inserted *NPP1* genes, respectively.

To gain insight into the function of NPP1, we produced and characterized mutants impaired in NPP1 function. Towards this end, a homozygous *npp1* knockout mutant possessing a *Tos17* retrotransposon in exon 5 of the genomic *NPP1* (Fig. 3A) was produced as described in the Materials and Methods. As shown in Fig. 3B and C, Southern blot analyses of genomic DNA from wild-type (WT) and *npp1* plants using radiolabeled *Tos17* and *NPP1* probes showed that *npp1* plants possess a single copy of *Tos17* inserted into the genomic *NPP1*.

**Fig. 4:**
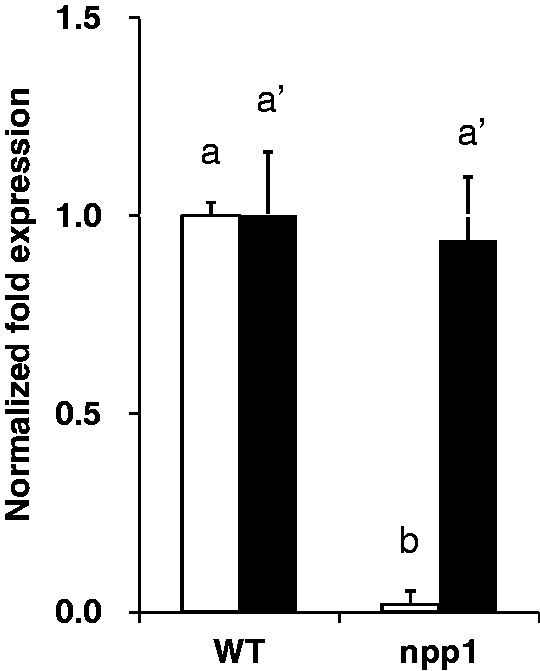
Complete loss of *NPP1* mRNA expression in shoots of the *npp1* mutant. The shoots of WT and *npp1* seedlings at 7 d after imbibition (DAI) were subjected to quantitation of *NPP1* (open squares) and *NPP6* (filled squares) mRNA levels. The ratio of *NPP* to *GAPDH* mRNA was normalized to 1 in the WT. The error bars represent the standard deviation in triplicate experiments. Columns with the same letter were not significantly different from each other (*P* ≥ 0.05, Student’s *t*-test).

To confirm the loss of function of *NPP1* in the *npp1* mutant, we analyzed the expression of *NPP1* mRNA and both the ADP-glucose pyrophosphatase (AGPPase) and UDP-glucose pyrophosphatase (UGPPase) activities. As illustrated in Fig. 4, the *NPP1* mRNA content was below detection limits in the *npp1* mutant shoots. In clear contrast, the expression of *NPP6* mRNA in the *npp1* mutant shoots was comparable with that of WT plants. It is noteworthy that AGPPase and UGPPase activities in *npp1* shoots were approximately 8% and 38% of that in WT shoots, respectively (Table 3), indicating that NPP1 is responsible for most of AGPPase and UGPPase activities in rice shoots.

**Table 3:** Down-regulation of *NPP1* expression is not accompanied by enhancement of AGPase, UGPase and SuSy activities

Enzyme activities (mU mg^−1^)	WT	*npp1*
AGPPase	95.6 ± 12.2	8.0 ± 1.0
AGPase	101.4 ± 10.0	89.5 ± 5.4
UGPPase	575.6 ± 70.3	216.8 ± 103.9
UGPase	587.4 ± 89.7	525.2 ± 68.2
Sucrose synthase	192.2 ± 24.3	205.1 ± 20.0

The shoots of WT and *npp1* seedlings at 7 d after imbibition were subjected to the enzyme assays.

Values show the means ± SD (*n* = 10). mU mg^−1^, nmol min^−1^ (mg of protein)^−1^.

### NPP1 exerts a negative effect on starch accumulation in shoots, especially under high CO_2_ concentrations and high temperature conditions

Nanjo et al. (2006) reported that NPP1 is from the ER–Golgi system to the chloroplast compartment through the secretory pathway in rice cells. As shown above, NPP1 catalyzes the hydrolytic breakdown of ADP-glucose (cf. Table 2). It also cleaves UDP-glucose, a nucleotide-sugar which is important for plant growth (Coleman et al. 2006, Coleman et al. 2009, Meng et al. 2009, Park et al. 2010, Zhang et al. 2011) and acts as the glucosyl donor for many transglucosylation reactions occurring in the Golgi apparatus, including those involved in the synthesis of cell wall polysaccharides (Staehelin and Moore 1995, Muñoz et al. 1996, Carpita 2011, Zhang et al. 2011). It is thus conceivable that NPP1 competes with enzymes involved in many transglucosylation reactions occurring in the Golgi apparatus and with starch synthase (SS) for the same UDP-glucose and ADP-glucose pools, respectively.

**Fig. 5:**
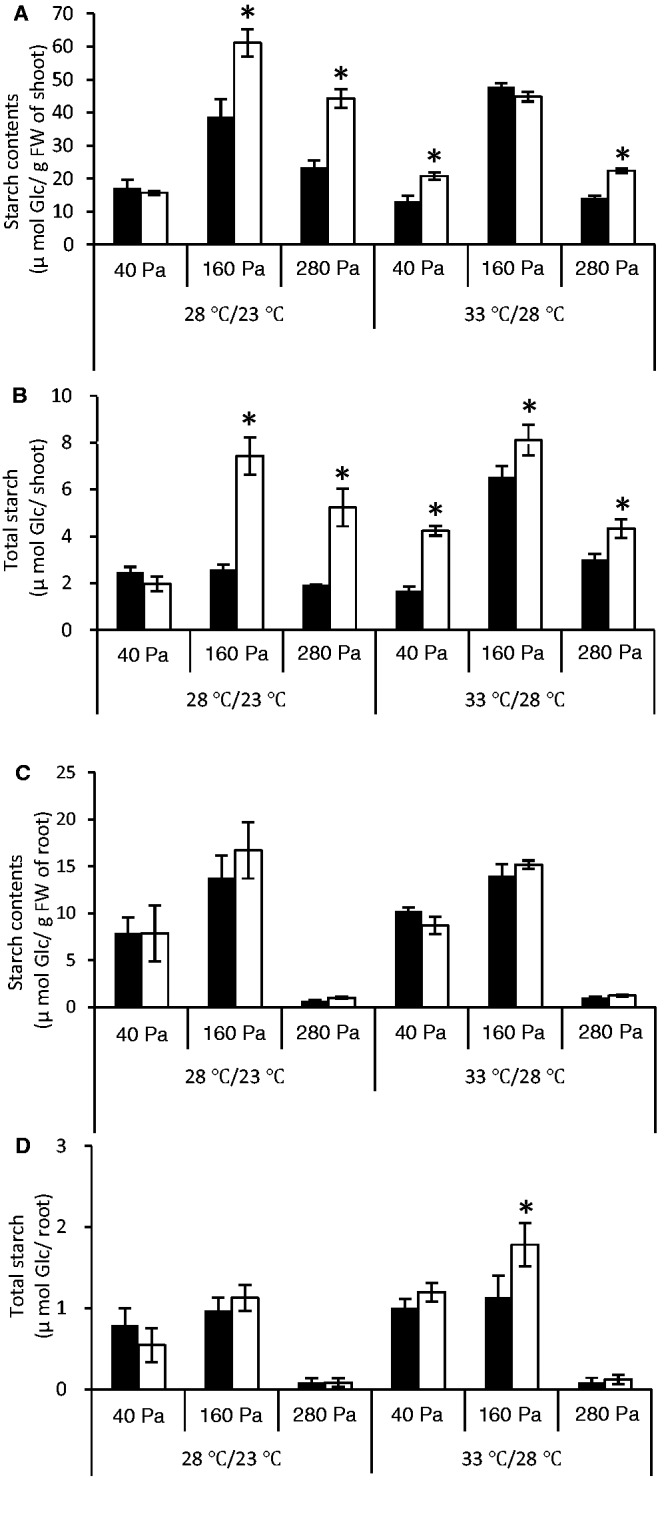
NPP1 exerts a negative effect on starch accumulation in rice shoots, especially under conditions of high temperature and CO_2_ concentrations. At 7 d after imbibition (DAI), WT (filled squares) and *npp1* (open squares) seedlings were further cultured for 7 d under different CO_2_ concentration (40–280 Pa) and temperature (12 h light/12 h dark: 28°C/23°C or 33°C/28°C) conditions. Results are presented as starch content per g FW of (A) shoots and (C) roots, or as total starch content in shoot and root (B and D, respectively). Values show the means ± SD (*n* = 4–10). Asterisks indicate significant differences by Student’s *t*-test (*P* > 0.05).

To investigate the possible involvement of NPP1 in starch metabolism, we compared the starch content between *npp1* and WT plants cultured under three different CO_2_ partial pressures: 40, 160 and 280 Pa (400, 1,600 and 2,800 p.p.m. concentration, respectively) and two temperature regimes: 28°C/23°C (12 h light/12 h dark) and ‘high temperature’ 33°C/28°C (12 h light/12 h dark) regimes. As shown in Fig. 5A and B, the starch content in *npp1* shoots was markedly higher than that of WT shoots when plants were cultured under high CO_2_ concentration conditions at any temperature regime. No difference in starch content could be found between *npp1* and WT shoots when plants were cultured at normal (40 Pa) CO_2_ under the 28°C/23°C (12 h light/12 h dark) temperature regime (Fig. 5A, B). In clear contrast, however, *npp1* shoots accumulated more starch than WT shoots when seedlings were cultured at the normal (40 Pa) CO_2_ concentration under the 33°C/28°C (12 h light/12 h dark) ‘high’ temperature regime. No significant differences in starch content could be found between roots of WT and *npp1* plants cultured under the various CO_2_ concentration and temperature conditions employed in this work (Fig. 5C, D).

Starch is considered the end-product of a pathway exclusively taking place in the plastid wherein AGPase is the sole enzyme that catalyzes the synthesis of ADP-glucose necessary for the SS-mediated reaction (Streb et al. 2009, Streb and Zeeman 2012). However, in recent years, an increasing volume of evidence has been provided that supports the occurrence of additional/alternative pathway(s) wherein sucrose synthase (SuSy) is also involved in the synthesis of ADP-glucose linked to starch biosynthesis (Baroja-Fernández et al. 2004, Muñoz et al. 2005, Baroja-Fernández et al. 2012; for a review, see Bahaji et al. 2013). Whether the increase in starch content in *npp1* shoots was due to a pleiotropic increase in enzymatic activities involved in ADP-glucose was investigated by comparing the maximum catalytic activities of ADP-glucose-producing enzymes (AGPase and SuSy) in shoots of WT and *npp1* plants. These analyses revealed that: (i) maximum AGPase and SuSy activities in rice shoots are comparable with those of AGPPase (Table 3); and (ii) no significant changes could be found in AGPase and SuSy activities between WT and *npp1* shoots (Table 3), indicating that the increase in starch content in the *npp1* shoots cannot be ascribed to enhanced activity of enzymes closely connected to ADP-glucose metabolism other than NPP1.

### Enhancement of *NPP1* expression results in reduced starch levels in mature leaves and in suspension-cultured cells of rice

The above data strongly indicated that NPP1 exerts a negative effect on starch accumulation in shoots, especially under high CO_2_ concentrations and high temperature conditions. Whether NPP1 is involved in the control of metabolic flux towards starch biosynthesis was further investigated by characterizing mature leaves from *NPP1*-overexpressing plants (UNP1) and mature leaves from *npp1* plants cultured at ambient CO_2_ concentration under a 28°C/23°C (14 h light/8 h dark) regime. As shown in Table 4, *NPP1* mRNA levels in UNP1 leaves were approximately 330% of that found in WT leaves. Furthermore, the UNP1 leaves exhibited a low starch phenotype, whereas the *npp1* leaves showed a high starch phenotype (Table 4).

**Table 4:** Starch contents in leaves of WT, *NPP1*-overexpressing UNP1 plants and *npp1* mutants

	NPP1 mRNA expression (normalized fold expression)	Starch contents (µmol Glc g^−1^ FW)
WT	1.00 ± 0.04	6.20 ± 1.37
UNP1	3.33 ± 0.71	3.97 ± 0.94
*npp1*	0.05 ± 0.05	12.00 ± 3.11

WT, UNP1 and *npp1* plants were grown for 52 d under the condition of 28°C/23°C (14 h light/8 h dark) and ambient CO_2_. Leaf samples harvested at the end of the day were subjected to starch assays.

Values show the means ± SD (*n* = 5).

We also characterized three independent lines of *NPP1*-overexpressing suspension-cultured cells (UNP1-1, UNP1-2 and UNP1-3). Suspension-cultured cells of rice actively synthesize and accumulate starch molecules. The cultured cells are frequently used as a model system for studying the biosynthesis and degradation of starch in rice. As shown in Table 5, the three lines exhibited high AGPPase activities when compared with WT cells. Furthermore, the ADP-glucose and starch contents in the three lines were significantly lower than those found in WT cells. No significant differences could be found in AGPase, SuSy, UDP-glucose pyrophosphorylase (UGPase), total phosphoglucomutase, total phosphoglucose isomerase, starch phosphorylase and hexokinase activities between the three *NPP1*-overexpressing lines and WT cells (Table 5), indicating that the reduction in starch content in UNP1 cells cannot be ascribed to changes in the enzyme activities other than AGPPase. Consistent with a previous report showing that up-regulation of AGPPase activity in potato plants results in pleiotropic enhancement of SS activity (Muñoz et al. 2008), total SS activities in the three *NPP1*-overexpressing lines were higher than in WT cells, which would indicate the possible occurrence of a fine regulation system preserving starch homeostasis in response to reduction of ADP-glucose content.

**Table 5:** Enzyme activities, ADP-glucose and starch contents in *NPP1*-overexpressing rice suspension-cultured cells

Enzyme activities (mU mg^−1^)	WT	UNP1-1	UNP1-2	UNP1-3
AGPPase	4.0 ± 0.3	204.4 ± 18.1	158.1 ± 12.4	113.1 ± 8.7
AGPase	126.2 ± 22.8	109.4 ± 11.7	96.1 ± 19.7	110.9 ± 12.5
SuSy	414.8 ± 25.6	387.2 ± 25.3	407.4 ± 45.3	257.5 ± 19.6
UGPase	206.7 ± 10.6	175.5 ± 14.3	233.1 ± 7.1	266.2 ± 15.0
Phosphoglucomutase	184.5 ± 5.0	167.1 ± 12.9	178.5 ± 17.5	176.9 ± 7.8
Phosphoglucoisomerase	129.1 ± 10.4	122.5 ± 16.4	125.5 ± 29.0	123.8 ± 9.8
Starch phosphorylase	102.8 ± 4.1	80.3 ± 5.1	97.6 ± 5.4	95.3 ± 10.5
Hexokinase	62.3 ± 5.5	78.6 ± 4.8	79.6 ± 12.3	69.1 ± 4.2
Soluble SS	50.4 ± 5.4	153.9 ± 17.7	151.6 ± 7.3	219.7 ± 33.9
ADP-glucose (nmol g^−1^ FW)	3.23 ± 0.21	1.02 ± 0.40	1.21 ± 0.13	1.51 ± 0.03
Starch contents (µmol glucose g^−1^ FW)	12.02 ± 0.23	5.35 ± 0.04	4.63 ± 0.04	3.52 ± 0.01

Suspension-cultured cells of WT and of three independent *NPP1*-overexpressing UNP1 lines were harvested after 7 d of culture and subjected to assays.

Values show the means ± sa (*n* = 3). mU mg^−1^, nmol min^−1^ (mg of protein)^−1^.

The overall data thus strongly indicated that NPP1 exerts a negative effect on starch accumulation, probably by competing with SS for the same ADP-glucose pool.

### NPP1 exerts a negative effect on growth of rice shoots and roots, especially under high CO_2_ concentrations and high temperature conditions

**Fig. 6:**
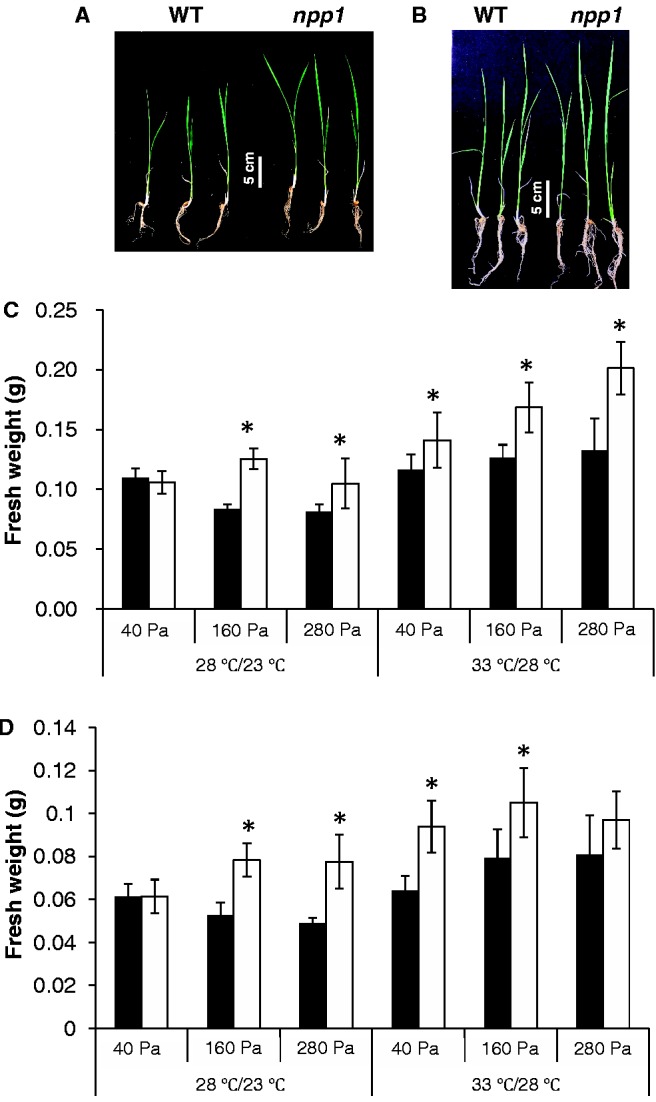
NPP1 exerts a negative effect on shoot and root growth, especially under conditions of high temperature and CO_2_ concentrations. At 7 d after imbibition, WT and *npp1* seedlings were incubated for an additional 7 d under different CO_2_ concentration (40–280 Pa) and temperature (12 h light/12 h dark: 28°C/23°C or 33°C/28°C) conditions. (A, B) Images of WT and *npp1* seedlings grown under 160 Pa CO_2_ at 28°C/23°C (A), and at 33°C/28°C (B). Graphics show the FW of dissected shoots (C) and roots (D) of WT (filled squares) and *npp1* (open squares) seedlings. Values show the means ± SD (*n* = 10). Asterisks indicate significant differences by Student’s *t*-test (*P* > 0.05).

Starch is a major determinant of plant growth (Sulpice et al. 2009), as demonstrated by studies of mutants that are defective in starch synthesis and/or mobilization (Caspar et al. 1985, Lin et al. 1988, Gibon et al. 2004). Because changes in NPP1 result in concomitant changes in starch content under high CO_2_ concentration conditions (Fig. 5, Tables 4, 5), and because changes in NPP1 also affect UGPPase activity (thus probably affecting the availability of UDP-glucose necessary for growth), we decided to explore the possible effect exerted by NPP1 on plant growth under different CO_2_ concentration conditions and temperature regimes. Towards this end, we compared the shoot and root FWs between WT and *npp1* plants. As shown in Fig. 6, shoots and roots of *npp1* plants were larger than those of WT plants at high CO_2_ concentrations (160 and 280 Pa) when plants were cultured under the 28°C/23°C (12 h light/12 h dark) regime. Shoots and roots of *npp1* plants were also larger than those of WT plants at normal atmospheric CO_2_ concentration (40 Pa) when cultured under the 33°C/28°C (12 h light/12 h dark) ‘high’ temperature regime (Fig. 6), the overall data thus strongly indicating that NPP1 exerts a negative effect on growth of rice shoots and roots, especially under high CO_2_ concentrations and high temperature conditions.

### Additional remarks

Taking into account all the limitations that are inherent in basing conclusions on genetically engineered plants, results presented in this work showing that NPP1 exerts a negative effect on both growth and starch accumulation in rice shoots provide the first in vivo evidence that, similar to mammalian NPPs, plant NPPs may play a role in regulatory aspects of plant metabolism by diverting carbon flux from one pathway to another according to biochemical and physiological needs. Also, results presented in this work provide evidence that down-regulation of NPP activity may represent a useful strategy for increasing starch content and biomass in crops. Needless to say, further studies will be necessary to confirm (or refute) this hypothesis.

Two different protein entities are responsible for the ADP-glucose hydrolytic activity in plants: ‘Nudix’ hydrolases (McLennan, 2006) and NPPs. Both Nudix and NPP AGPPases have been shown to occur in the plastidic compartment (Nanjo et al. 2006, Muñoz et al. 2008). Therefore, it is highly conceivable that these enzymes compete with SS for the same ADP-glucose pool, thus preventing starch biosynthesis. Consistent with this view, Muñoz et al. (2008) reported that up-regulation of the plastidic Nudix AGPPase in transgenic potato plants resulted in reduced levels of starch content in both leaves and tubers (Muñoz et al. 2008). It is noteworthy that the results presented in Fig. 4 and Table 3 showing that shoots of *npp1* rice plants impaired in the NPP1 function possess approximately 8% of the WT AGPPase activity and accumulate higher starch content than WT shoots would indicate that NPP1: (i) is a major determinant of AGPPase activity; and (ii) prevents metabolic flux towards starch in rice plants by reducing the plastidic pool of ADP-glucose linked to starch biosynthesis.

Maximal in vitro AGPase activity greatly exceeds the minimum required to support the normal rate of starch accumulation in some organs (Denyer et al. 1995, Weber et al. 2000, Li et al. 2013). The flux control coefficient of AGPase can be as low as 0.08 (Denyer et al. 1995, Weber et al. 2000, Rolletschek et al. 2002) and, thus, AGPase is not a rate-limiting step in starch biosynthesis in some organs. In contrast, the maximum catalytic activity of SS is close to metabolic flux through starch biosynthesis in several organs. Furthermore, SS can have a starch biosynthesis flux control coefficient approaching 1 when plants are exposed to SS non-inactivating temperature (Keeling et al. 1993), indicating that SS is a major site of regulation of starch synthesis under SS non-inactivating temperature conditions. It is noteworthy that the results presented in Fig. 5A and B showing that starch content in *npp1* shoots is higher than in WT shoots at normal (400 p.p.m.) CO_2_ concentration conditions under the ‘high temperature’ regime, but not under the ‘low temperature’ regime, would indicate that, under normal CO_2_ concentration and ‘high temperature’ conditions, NPP1 is a major site of regulation of starch synthesis. This is probably because under ‘high temperature’ conditions: (i) ADP-glucose hydrolytic NPP1 activity increases to levels exceeding those of AGPase and/or SS; and/or (ii) AGPase and/or SS activities decrease far below that of ADP-glucose hydrolytic NPP1 activity at normal CO_2_ concentration and ‘high temperature’ conditions.

Rice NPPs catalyze the hydrolytic breakdown of pyrophosphate and phosphodiester bonds of a number of nucleotides and nucleotide-sugars (Table 2; Kaneko et al. 2011). These include APS, which acts not only as the precursor molecule for the synthesis of essential amino acids and as substrate for sulfation reactions (Schmidt and Jäger 1992, Leustek 1996), but also as a potent competitive inhibitor of enzymes involved in sulfur metabolism (Renosto et al. 1993). Also, rice NPPs efficiently recognize ADP-ribose as substrate (Table 2). This nucleotide-sugar is produced enzymatically as part of the turnover of NAD^+^, cyclic ADP-ribose and poly(ADP)-ribose. Because of its free aldehydic group, high intracellular ADP-ribose levels can result in non-enzymatic ADP-ribosylation of proteins, a deleterious process that inactivates enzymes and interferes with recognition processes that rely on enzymatic ADP-ribosylation (Jacobson et al. 1997). Non-enzymatic glycation of long-lived proteins by reducing sugars increases with age, producing what have been called advanced glycosylation end-products, which may be targets for degradation and thereby trigger apoptosis in eukaryotic cells (Kaneto et al. 1996). Therefore, similarly to some plant Nudix hydrolases preventing damage by regulating the intracellular concentrations of reactive nucleotides under biotic and abiotic stress conditions (Ishikawa et al. 2009, Ogawa et al. 2009, Briggs and Bent 2011), plant NPPs may play a role in sanitizing the metabolic pool of potentially harmful nucleotides. Needless to say, further investigations will be necessary to confirm (or refute) this hypothesis.

## Materials and Methods

### Plant materials

The rice variety used in this study was *Oryza sativa* L. cv. Nipponbare. A *Tos17*-inserted line of *NPP1* (ND8012) was obtained from the National Institute of Agrobiological Sciences (NIAS, Tsukuba, Japan; Miyao et al. 2003). After backcrossing twice with the WT plants and self-pollination, screening was carried out by Southern blotting for homozygous *npp1* plants. *Tos17* and *NPP1* were amplified by PCR with the appropriate primer sets (Supplementary Table S3), and ^32^P-labeled using the Rediprime II DNA Labeling System (GE Healthcare). Total DNA from rice leaves was digested with *Xba*I, fractionated by agarose gel electrophoresis, alkali-transferred to Hybond-N+, hybridized for 16 h at 65°C with the ^32^P-labeled *Tos17* and *NPP1* probes, washed under high stringency conditions and exposed to IP (Fuji film). The exposed IP was scanned by BAS-5000 (Fuji film).

Rice calli derived from the embryo portions of seeds were cultured as described by Mitsui et al. (1996). Callus cells were grown in a Sakaguchi flask in Murashige and Skoog (MS) medium containing 3% (w/v) sucrose, 2 mg l^−1^ 2,4-D and 5 mg l^−1^ thiamine-HCl, placed on a reciprocal shaker operated at 110 strokes min^−1^ with 70 mm amplitude, at 28°C in darkness. The established suspension-cultured cells were subcultured at 7 d intervals. All these procedures were performed under aseptic conditions.

### Cloning of rice NPP encoding cDNAs

A complete rice *NPP1* cDNA was cloned from rice shoot cDNA libraries as described by Nanjo et al. (2006). Using the *NPP1* cDNA as a radiolabeled probe, we screened the shoot cDNA libraries to isolate other NPP-encoding cDNAs. The complete cDNAs thus obtained, designated as *NPP2*, *NPP3*, *NPP4*, *NPP5* and *NPP6*, were cloned into pBluescript SK(–) (Stratagene) to create pOsNPP2, pOsNPP3, pOsNPP4, pOsNPP5 and pOsNPP6.

### Plasmid constructs

Plasmids used in this study and references describing how they were constructed are listed in Supplementary Table S4. For overexpression of rice *NPP2* and *NPP6* in rice plants and cultured cells, *Bam*HI–*Kpn*I PCR fragments were amplified from pOsNPP2 and pOsNPP6, and cloned into the *Bam*HI and *Kpn*I sites of the p2K-1+ plant expression vector (Christensen et al. 1992, Miki et al. 2004) to produce p2K-Ubi-OsNPP2 and p2K-Ubi-OsNPP6, respectively.

### Genetic transformation

The binary vectors were incorporated into competent cells of *Agrobacterium tumefaciens* strain EHA101 (Hood et al. 1986) and treated with 20 mM CaCl_2_. *Agrobacterium*-mediated transformation and regeneration of rice plants were performed according to the methods described by Hiei et al. (1994). Cultured rice cells were grown in hygromycin selective medium for 2 weeks and then transferred to a redifferentiative medium for 1 month. The following transgenic rice lines were established: UNP1, UNP2 and UNP6 transformed with p2K-Ubi-OsNPP1, p2K-Ubi-OsNPP2 and p2K-Ubi-OsNPP6, respectively.

### Gene expression analyses

Measurements of mRNA contents were performed by reverse transcription–PCR (RT–PCR) and by Northern blot analyses. For RT–PCR analyses, total RNA was extracted from each mutant or transgenic rice plant using an RNeasy Plant Mini Kit (Qiagen). First-strand cDNA synthesis was carried out via a TaKaRa RNA PCR™ Kit (AMV) Ver.3.0 (TAKARA) with an Oligo dT-Adaptor Primer. PCR was performed with a set of forward and reverse primers as shown in Supplementary Table S5. Northern blotting was performed according to the procedure described previously (Kashem et al. 2000, Nanjo et al. 2004). The DNA probes specific for *NPP1*, *NPP2* and *NPP6* were amplified by PCR with primer sets (Supplementary Table S6). The DNA probe for rice actin 1 was prepared as described earlier (Kashem et al. 2000).

### Determination of starch content

Measurements of starch content were carried out as follows. Harvested shoots and roots were immediately frozen and powdered in liquid nitrogen using a pestle and mortar. Samples were homogenized in 5 vols. of 80% (v/v) ethanol, boiled for 1 min and centrifuged at 16,000 × *g* for 10 min at 4°C. The pellets including starch were re-extracted with 80% ethanol four times and dried using a SpeedVac. The dried pellets were resuspended in dimethylsulfoxide and boiled for 30 min, then centrifuged at 16,000 × *g* for 10 min at room temperature. An aliquot of the collected supernatant was incubated in 50 mM acetate buffer (pH 5.0) with 44 U ml^−1^ α-amylase and 28 U ml^−1^ amyloglucosidase at 37°C overnight. The reaction mixture was further incubated in 260 mM Tris–HCl (pH 7.6) with 4 mM MgCl_2_, 1.3 mM ATP, 0.78 mM β-NAD^+^, 3.9 U ml^−1^ hexokinase and 1.3 U ml^−1^ glucose-6-phosphate dehydrogenase at 37°C for 30 min. The amount of glucose resulting from starch was determined spectrophotometrically by measuring the absorbance of NADH at 340 nm.

### Determination of ADP-glucose content

Frozen tissues (600 mg) were ground with liquid nitrogen and homogenized with 5 ml of 10% (w/v) trichloroacetic acid (TCA). The homogenates were centrifuged at 10,000 × *g* for 10 min and the collected supernatant was extracted with diethyl ether to remove TCA, and lyophilized. The dried samples were suspended with 0.5 ml of water and passed through a solid-phase extraction cartridge (Sep-Pak C18, Waters). Contents of ADP-glucose in the flow-through fraction were determined employing reverse-phase ion-pairing liquid chromatography coupled to mass spectrometry described by Lu et al. (2010). A liquid chromatograph–mass spectrometer for nucleotide-sugar identification and quantitation consists of an LaChrom Elite-HPLC system with an L-2130 pump (Hitachi) and LTQ Orbitrap XL (ThermoFisher Scientific) controlled by Xcalibur 2.0 software. Liquid chromatography separation was carried out on a Hypersil GOLD column (50 × 2.1 mm, 5 µm particle size, ThermoFisher Scientific), using reversed-phase chromatography with tributylamine in the aqueous mobile phase to enhance retention and separation. An aliquot of sample (10 µl) was loaded onto the Hypersil GOLD column equilibrated with solvent A at a flow rate of 150 µl min^−1^. Solvent A was 97 : 3 water : methanol with 10 mM tributylamine and 15 mM acetic acid; solvent B was methanol. The gradient is: 0 min, 0% B; 2.5 min, 0% B; 5 min, 20% B; 7.5 min, 20% B; 13 min, 55% B; 15.5 min, 95% B; 18.5 min, 95% B; 19 min, 0% B; 25 min, 0% B. Other liquid chromatography parameters are autosampler temperature 4°C, injection volume 10 µl and column temperature 30°C. Various instrumental settings were optimized as follows: sheath gas flow rate 25 (arbitrary units) and sweep gas flow rate 0 (arbitrary units), spray voltage 2.5 kV, capillary temperature 300°C. The instrument was mass calibrated using the polytyrosine-1,3,6 standards every 3 d.

### Enzymatic activity and protein assays

Harvested tissues were immediately ground to a fine powder in liquid nitrogen with a pestle and mortar. A 1 g aliquot of the frozen powder was suspended with 5 ml of extraction buffer consisting of 100 mM HEPES (pH 7.5), 2 mM EDTA and 5 mM dithiothreitol at 4°C. The suspension was desalted and assayed for enzymatic activities. We verified that this procedure did not result in loss of enzymatic activity by comparing activity in extracts prepared from the frozen powder with extracts prepared by homogenizing fresh tissue in extraction medium. The methods of measurement of starch phosphorylase (Mu et al. 2001), soluble SS (Jiang et al. 2003, Nishi et al. 2001), UGPase (Kimura et al. 1992), hexokinase (Guglielminetti et al. 2000), phosphoglucomutase (Davies et al. 2003) and phosphoglucoisomerase (Kelley et al. 1984) activities were described previously. AGPase was measured following the two-step assay method described by Li et al. (2012). ADP-glucose producing SuSy activity was measured as described by Baroja-Fernández et al. (2012). AGPPase, UGPPase and other nucleotide-sugar- and nucleotide-hydrolyzing NPP activities were measured as described by Rodríguez-López et al. (2000).

Protein contents were determined by the procedure of Bradford (1976).

### Purification of NPPs

Purification procedures of NPP1, NPP2 and NPP6 expressed in the transgenic rice cells (UNP1, UNP2 and UNP6, respectively) were essentially identical to those used in the earlier study (Nanjo et al. 2006). They are summarized in Supplementary Table S2. Rice callus cells (200 g) derived from the seed embryos of UNP1, UNP2 and UNP6 lines were homogenized in 5 vols. of 10 mM Tris–HCl (pH 8.8) and filtered through four layers of gauze. The homogenates were centrifuged at 20,000 × *g* for 10 min. The supernatants were then adjusted to acidic pH 5.4 with acetate buffer and centrifuged at 20,000 × *g*. After re-adjustment to pH 7.4 with 1.5 M Tris–HCl (pH 8.8) and further centrifugation at 20,000 × *g*, the resulting supernatants were applied to a Con A–Sepharose 4B column (ø 1.0 × 8 cm, Pharmacia) equilibrated with 40 mM Tris–HCl (pH 7.4), 0.5 M NaCl, 1 mM MnCl_2_ and 1 mM CaCl_2_, and eluted with 25 ml of 0.5 M α-methyl-d-mannopyranoside in 10 mM Tris–HCl (pH 7.4). The eluents were applied to a Q Sepharose HP column (ø 1.0 × 3 cm, Pharmacia) equilibrated with 10 mM Tris–HCl (pH 7.4) and eluted with 50 ml of a linear gradient of 0–0.5 M NaCl in 10 mM Tris–HCl (pH 7.4). Finally, the enzyme preparation was desalted by ultrafiltration on a Microcon YM-100 (Amicon).

### Accession numbers

Rice NPP genes whose complete cDNAs were isolated were: *NPP1* (AB100451, AK072408), *NPP2* (AB196673), *NPP3* (AK101976), *NPP4* (AK073512), *NPP5* (AK121432) and *NPP6* (AK102346).

## Supplementary data

Supplementary data are available at PCP online.

## Funding

This research was supported by the Ministry of Education, Culture, Sports, Science, and Technology, Japan [Scientific Research on Innovative Areas (22114507) and Grants-in-Aid for Scientific Research (B) (22380186)]; the Comisión Interministerial de Ciencia y Tecnología and Fondo Europeo de Desarrollo Regional (Spain) [grant BIO2010-18239]; the Government of Navarra [grant IIM010491.RI1].

## Supplementary Material

Supplementary Data

## Abbreviations

AGPase: ADP-glucose pyrophosphorylase
APS: adenosine 5′-phosphosulfate
ER: endoplasmic reticulum
GFP: green fluorescent protein
NPP: nucleotide pyrophosphatase/phosphodiesterase
RT–PCR: reverse transcription–PCR
SS: starch synthase
SuSy: sucrose synthase
UGPase: UDP-glucose pyrophosphorylase
UGPPase: UDP-glucose pyrophosphatase
WT: wild type
