# Supplementary material for: Nucleotide Pyrophosphatase/Phosphodiesterase 1 Exerts a Negative Effect on Starch Accumulation and Growth in Rice Seedlings under High Temperature and CO2 Concentration Conditions
Source: Plant Cell Physiol. 2013 Oct 21;55(2):320–32. doi: 10.1093/pcp/pct139 (PMC3913438; doi:10.1093/pcp/pct139)
Supplement: Supplementary Data [file supp_55_2_320__index.html]

Nucleotide Pyrophosphatase/Phosphodiesterase 1 Exerts a Negative Effect on Starch Accumulation and Growth in Rice Seedlings under High Temperature and CO2 Concentration Conditions — Nucleotide Pyrophosphatase/Phosphodiesterase 1 Exerts a Negative Effect on Starch Accumulation and Growth in Rice Seedlings under High Temperature and CO2 Concentration Conditions — Nucleotide Pyrophosphatase/Phosphodiesterase 1 Exerts a Negative Effect on Starch Accumulation and Growth in Rice Seedlings under High Temperature and CO2 Concentration Conditions — Supplementary Data 

# Nucleotide Pyrophosphatase/Phosphodiesterase 1 Exerts a Negative Effect on Starch Accumulation and Growth in Rice Seedlings under High Temperature and CO2 Concentration Conditions

## Supplementary Data

files

**Files in this Data Supplement:**

- Supplementary Data - pdf file
