# Supplementary material for: Nucleotide Pyrophosphatase/Phosphodiesterase 1 Exerts a Negative Effect on Starch Accumulation and Growth in Rice Seedlings under High Temperature and CO2 Concentration Conditions
Source: Plant Cell Physiol. 2013 Oct 21;55(2):320–32. doi: 10.1093/pcp/pct139 (PMC3913438; doi:10.1093/pcp/pct139)
Supplement: Supplementary Data [file supp_pct139_pcp-2013-e-00369-File008.pdf]

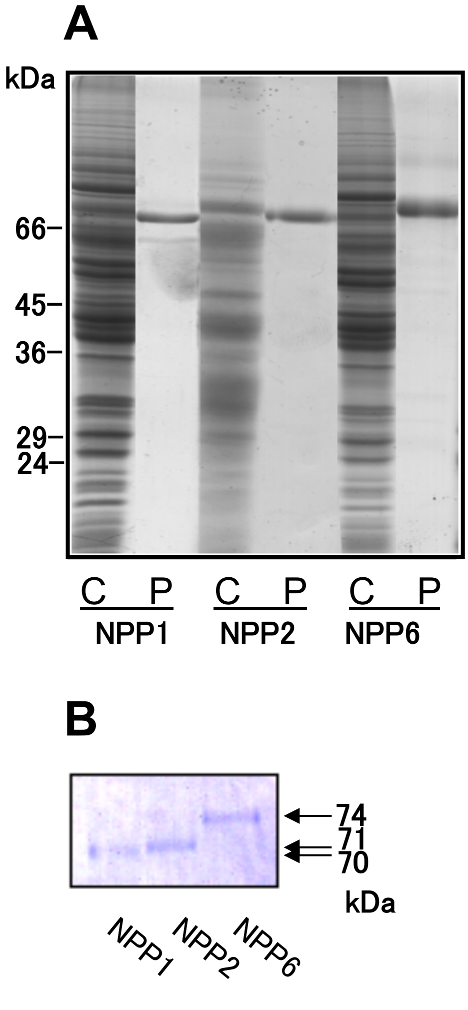

Supplemental Fig. S1 Purification of NPP1, NPP2 and NPP6.

NPP1, 2 and 6 proteins were purified from the transgenic rice lines UNP1, 2 and 6 by employing Con A-Sepharose column chromatography (see Supplemental Table 2), and subjected to SDS-PAGE, followed by Coomassie Blue staining. In panel A, left (C) and right lanes (P) represent crude and purified enzyme preparations, respectively. Molecular size markers in kDa are shown on the left. As shown in panel B, the molecular species of NPP were distinguished by their migration on the SDS-gels.

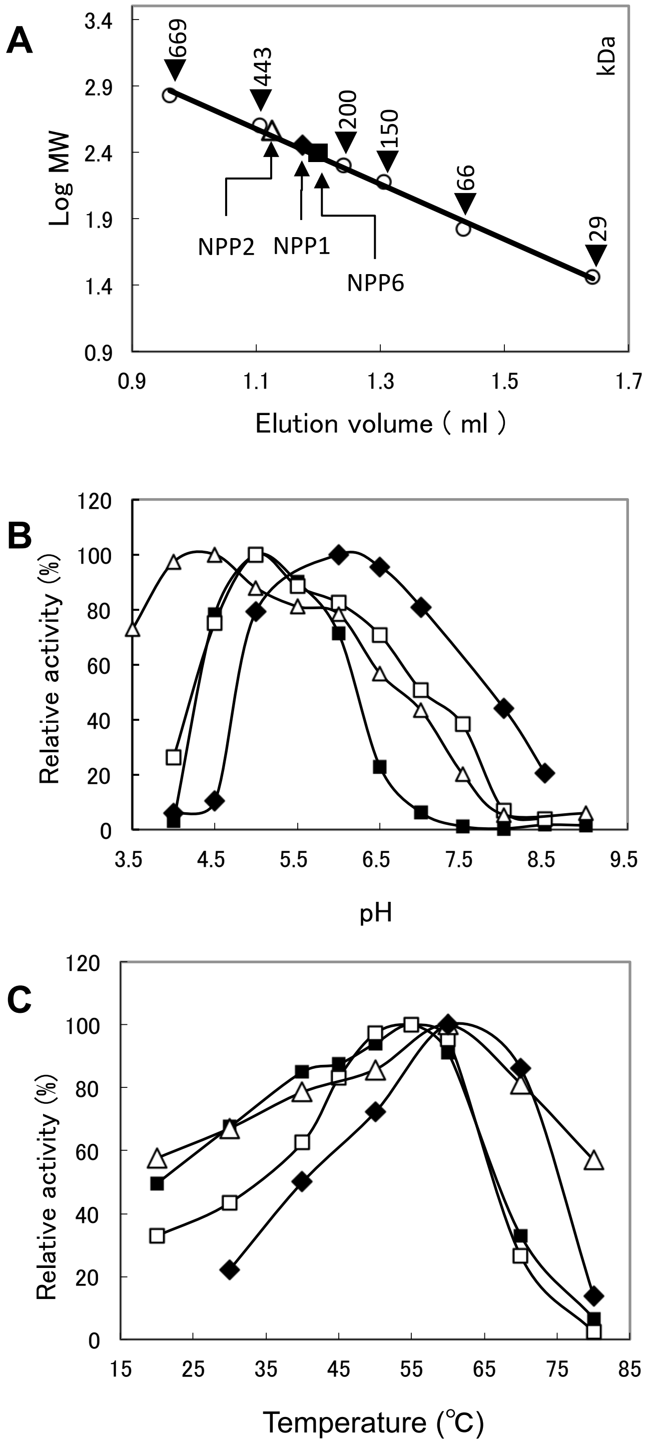

Supplemental Fig. S2 Determination of molecular sizes, optimum pH and temperatures of NPP1, 2 and 6.

(A) Molecular sizes of purified NPP1, 2 and 6 proteins were estimated by gel filtration chromatography with molecular weight standards, thyroglobulin (669 kDa), apoferritin (443 kDa), β-amylase (200 kDa), alcohol dehyrogenase (150 kDa), albumin bovine serum (66 kDa) and carbonic anhydrase (29 kDa). **(B)** Effects of pH on the enzyme reaction of NPP1 (AGPPase), 2 (ADPase) and 6 (AGPPase and ADPase). Citrate-NaOH (pH 3.5), acetate-NaOH (pH 4.0-5.0), MES-NaOH (pH 5.5-6.5), HEPES-NaOH (pH 7.0-7.5), Tris-HCl (pH 8.0-9.0) and Glycine-NaOH (pH 10.0) were used in the assays. **(C)** Effects of temperature on the enzyme reaction of NPP1 (AGPPase), 2 (ADPase) and 6 (AGPPase and ADPase). Filled diamonds, NPP1 AGPPase; open triangles, NPP2 ADPase; filled squares, NPP6 AGPPase; open squares, NPP6 ADPase.

Supplemental Table S1.

Genetic information and characteristics of the deduced proteins of *NPPs*

	Accession No.	Locus	Position	Nucleotide Length (bp) of cDNA	ORF (bp)	Amino Acid Length	Molecular Weight	Isoelectric Point
NPP1	AB100451 (AK072408)	Os08g0531000	chr8:26432389 ..26438231 (+ strand)	2201	1872	623	69878.88	5.85
NPP2	AB196673	-	chr12:23852792 ..23856588 (- strand)	2347	1854	617	69559.30	6.08
NPP3	AK101976	Os03g0214000	chr3:5941413 ..5946570 (+ strand)	2093	1848	615	68133.25	5.86
NPP4	AK073512	Os12g0576700	chr12:23844245 ..23847990 (- strand)	2019	1836	611	68800.99	5.85
NPP5	AK121432	Os12g0576600	chr12:23838779 ..23842486 (- strand)	2237	1824	607	68474.70	6.20
NPP6	AK102346	Os09g0506000	chr9:19286496 ..19291215 (- strand)	2322	1893	630	69403.44	6.02

Supplemental Table S2

Table 2a. Purification of NPP1 from cultured cells of UNP1

	Total Volume (mL)	Total Protein (mg)	Total Activity (U^a)	Specific Activity (U/mg prot.)	Purification (fold)
Crude Extract	500	1945.44	339.28	0.17	-
pH5.4	500	1645.42	327.22	0.199	1.14
Con A-Sepharose 4B	30	1.76	52.60	29.92	171.57
Q Sepharose HP	5	0.35	15.05	42.24	242.23
Microcon YM-100	1	0.03	3.21	108.83	624.05

[a] $\mu mol\ min^{-1}$

Table 2b. Purification of NPP2 from cultured cells of UNP2

	Total Volume (mL)	Total Protein (mg)	Total Activity (U^a)	Specific Activity (U/mg prot.)	Purification (fold)
Crude Extract	500	742.24	153.44	0.206	-
Con A-Sepharose 4B	30	1.58	16.65	10.57	51.15
Q Sepharose HP	5	0.75	8.20	10.88	52.62
Microcon YM-100	1	0.25	2.81	11.13	53.83

[a] $\mu mol\ min^{-1}$

Table 2c. Purification of NPP6 from cultured cells of UNP6

	Total Volume (mL)	Total Protein (mg)	Total Activity (U^a)	Specific Activity (U/mg prot.)	Purification (fold)
Crude Extract	500	837.55	251.70	0.30	-
pH 5.4	500	576.60	206.88	0.36	1.19
Con A-Sepharose 4B	30	2.99	16.96	5.67	18.87
Q Sepharose HP	5	0.60	11.29	18.67	62.14
Microcon YM-100	1	0.08	3.52	43.54	144.86

[a] $\mu mol\ min^{-1}$

Supplemental Table S3.

Screening *Tos17*-inserted mutants of rice

Target DNA	Primer sequences
Tos17	
	forward: 5‘-tgaagcatcggtctcagcta-3’
	reverse: 5’-gtaggttgggagggttgtga-3’
NPP1	
	forward: 5’-ccggcacgcactgttgggt-3’
	reverse: 5’-cgccgaagacgatgatgcg-3’

Supplemental Table S4.

Overexpression of rice *NPP1, 2* and *6* in rice plants

Constructed plasmids	DNA template	Primer sequences
p2K-Ubi-OsNPP1 (Nanjo et al., 2006)		
p2K-Ubi-OsNPP2	pOsNPP2	5'-agggatcccatgaggttcttgactatggca -3'<br>5'-atggtacctcatactgccaaggtggt-3'
p2K-Ubi-OsNPP6	pOsNPP6	5'-atggatccatggcgatgccgctgggtggt-3'<br>5'-aaggtacctcacgtcgccggcgggat-3'

Supplemental Table S5.

Quantitative RT-PCR

Target DNA	Primer sequences
NPP1 (AB100451)	
	forward: 5’-gagttgatgcctcatcgtc-3'
	reverse: 5’- aagcgatcgaccactaccac-3’
NPP6 (AK102346)	
	forward: 5’-gtcccctagaaaaacccact-3'
	reverse: 5’-gcactctttggcggtatcc-3’
GAPDH (AK070032)	
	forward: 5’-cgccaagcactgatttgttgaaa-3'
	reverse: 5’-gttccgcttgcccaggtc-3’

Supplemental Table S6.

Preparation of DNA probes for Northern blotting

Target DNA	Primer sequences
NPP1 (AB100451)	
	forward: 5’-ggcgaactacgtgtactgg-3'
	reverse: 5’- gatcgaccgaccgatcactc-3’
NPP2 (AB196673)	
	forward: 5’-tcatatggtgaaccaatgggc-3'
	reverse: 5’-ccagctccactgtcactata-3’
NPP6 (AK102346)	
	forward: 5’-ggcggcggcgtgtgggcgtt-3'
	reverse: 5’-gctgtgcgcgtaggagggctcc-3’
